# Endothelial selective adhesion molecule and interleukin-16 play an intermediary role in psoriasis complicated with acute myocardial infarction: A Mendelian randomization study

**DOI:** 10.1097/MD.0000000000042538

**Published:** 2025-05-23

**Authors:** Xuan Kang, Chao Wang, Guo-Qiang Zhong, Hai-Jun Zhang

**Affiliations:** a Department of Endocrinology and Metabolism, The First Affiliated Hospital, Hengyang Medical School, University of South China, Hengyang, Hunan Province, China; b Department of Cardiology, The First Affiliated Hospital of Guangxi Medical University, Nanning, Guangxi Province, China; c Department of Cardiology, The First Affiliated Hospital, Hengyang Medical School, University of South China, Hengyang, Hunan Province, China.

**Keywords:** acute myocardial infarction, endothelial cell-selective adhesion molecule, interleukin-16, Mendelian randomization, psoriasis

## Abstract

Psoriasis is a prevalent inflammatory skin disorder, often associated with an increased risk of atherosclerosis. Despite growing evidence suggesting a potential link between psoriasis and acute myocardial infarction (AMI), the causal relationship remains uncertain and is still a subject of debate. This study aims to fill this knowledge gap by utilizing Mendelian randomization (MR) and mediation analysis to systematically evaluate the causal association between psoriasis and AMI. Additionally, we seek to identify potential mediators that may influence this relationship, thereby providing new insights into the underlying mechanisms that could explain the observed association. The psoriasis GWAS dataset (2802 cases, 212,242 controls) was obtained from the FinnGen study, while genetic associations with endothelial selective adhesion molecule (ESAM) and interleukin (IL)-16 levels were derived from meta-analyses by Sun et al (3301 individuals) and Ahola-Olli et al (3483 individuals), respectively. AMI outcome data (3927 cases, 333,272 controls) were extracted from the UK Biobank. Two-sample MR analyses were conducted to assess the causal effects of psoriasis, ESAM, and IL-16 on AMI risk. MR mediation analysis was used to determine whether ESAM and IL-16 mediate the effect of psoriasis on AMI. To minimize the impact of population differences, we employed robust genetic instruments (*F* > 10) for each exposure, conducted sensitivity analyses (MR-Egger and weighted median) to check for pleiotropy, and ensured the validity of our results across different populations. The genetic liability to psoriasis (odds ratio [OR]: 1.00078; 95% confidence interval [CI]: 1.00008–1.00148; *P *= .028479), ESAM (OR: 1.00208; 95% CI: 1.00019–1.00397; *P *= .031089), and IL-16 (OR: 1.00118; 95% CI: 1.00009–1.00227; *P *= .033826) were associated with higher AMI risks. The proportion of the effects of genetically-predicted psoriasis mediated through genetically-predicted ESMA and IL-16 was 24.8% (95% CI 8.3%–41.2%) and 16.1% (95% CI 5.3%–26.8%), respectively. Genetic liability to psoriasis is correlated with a higher risk of AMI, which is partially mediated by ESAM and IL-16. The targeted intervention of ESAM and IL-16 might help decrease the risk of AMI in psoriasis patients.

## 1. Introduction

Acute myocardial infarction (AMI) has become one of the leading causes of hospitalization and mortality worldwide, posing a significant threat to global public health. Atherosclerosis, the initial step in the development of AMI, is fundamentally a chronic inflammatory process that involves multiple immune and inflammatory mechanisms.^[1]^ Psoriasis, a common inflammatory skin disorder, has been increasingly recognized as a condition that can elevate the risk of atherosclerosis by approximately 25%, independent of traditional risk factors such as obesity, smoking, and hyperlipidemia.^[2]^ Notably, psoriasis patients with a disease duration exceeding 8 years are at a significantly higher risk of developing atherosclerosis compared to healthy individuals.^[3]^ Furthermore, atherosclerosis serves as the underlying pathology for psoriasis patients who are predisposed to cardiovascular diseases. Both atherosclerosis and psoriasis share numerous overlapping inflammatory pathways, such as systemic and local immune responses, as well as the activation of chemokines and cytokines, including S100 calcium-binding protein (S100) A8/A9, tumor necrosis factor-α (TNF-α), vascular endothelial growth factor (VEGF), interleukin (IL)-12/17A, and monocyte chemotactic protein 1 (MCP-1).^[4]^

In addition to these inflammatory mechanisms, the endothelial cell-selective adhesion molecule (ESAM), a member of the immunoglobulin superfamily, has been shown to play a pivotal role in atherosclerosis. It regulates endothelial permeability, tight junctions, and angiogenesis, while promoting the migration of neutrophils and monocytes to sites of vascular injury.^[5–7]^ Furthermore, several inflammatory mediators involved in atherosclerosis, such as E-selectin, C-C motif chemokine ligand 7 (CCL7), and IL-16, have been correlated with the severity of atopic dermatitis, which shares pathophysiological features with psoriasis.^[8]^ Studies have demonstrated that IL-16 is also implicated in the inflammatory processes of AMI, with a direct link to inflammatory cell activation.^[9]^ However, the direct causal relationship between psoriasis, ESAM, IL-16, and AMI remains poorly understood. Furthermore, the potential mediating roles of ESAM and IL-16 in psoriasis-induced AMI susceptibility are yet to be explored in depth.

The interaction between the genome, epigenome, and environment is increasingly recognized as a significant driver of complex diseases such as psoriasis, atherosclerosis, and AMI. The epigenome, encompassing DNA methylation, histone modifications, chromatin remodeling, and regulatory RNAs (including microRNAs and long noncoding RNAs), interacts with environmental factors – such as diet, pathogens, and climate – to influence gene expression and phenotype development.^[10,11]^ Multi-level interactions between genetic, epigenetic, and environmental factors play a critical role in disease onset and progression, with growing evidence linking epigenetic variations to disease outcomes.^[12]^ Gene expression is tightly regulated in both space and time, with distinct gene subsets being activated in different tissues and developmental stages.^[13–15]^ Furthermore, the quantity of gene products within tissues impacts overall gene expression, making the study of gene regulation at the cellular and chromosomal levels fundamental to understanding disease processes.^[16]^

Given the complexity of these molecular networks, it is essential to utilize analytical methods that can reliably disentangle causal relationships from confounding factors. Mendelian randomization (MR) has emerged as a powerful tool to infer causality by using genetic variations as instrumental variables.^[17]^ Since genetic variations are randomly assigned at conception, their associations with disease outcomes are less susceptible to environmental confounders. MR has been successfully employed to identify mediating pathways in various diseases^[18,19]^; however, to the best of our knowledge, the causal relationships between psoriasis, ESAM, IL-16, and AMI have yet to be explored using this method. This study aims to fill this critical knowledge gap by evaluating the causal effects of psoriasis, ESAM, and IL-16 on the risk of AMI through the MR framework. Specifically, we employ MR mediation analysis to assess whether ESAM and IL-16 mediate the relationship between psoriasis and AMI risk. This research is the first to apply MR and MR mediation analysis to this issue, providing novel insights into how these factors contribute to AMI risk. Compared to other studies, this approach has significant advantages: it uses robust genetic instruments to minimize confounding, utilizes large-scale GWAS datasets, and comprehensively examines the complex interplay between psoriasis, ESAM, IL-16, and AMI, which has not been sufficiently addressed in prior research. This methodology strengthens the reliability of our conclusions and offers a more detailed understanding of the underlying mechanisms involved.

## 2. Methods

### 2.1. Data sources

The genome‐wide association studies (GWAS) dataset of psoriasis including 2802 cases and 212,242 controls was extracted from FinnGen (https://www.finngen.fi/fi) study. The genetic associations with ESAM were obtained from the GWAS meta-analysis reported by Sun et al.^[20]^ This study quantified 3622 plasma proteins including ESAM protein based on 3301 healthy subjects extracted from the INTERVAL study. The GWAS dataset of IL-16 levels was obtained from the GWAS meta-analysis reported by Ahola-Olli et al.^[21]^ This study quantified the IL-16 levels based on 3483 healthy subjects extracted from the 3 independent Finnish population cohorts. The outcome data of AMI, including 3927 cases and 333,272 controls, were extracted from the United Kingdom Biobank supported by the Neale Lab Consortium (https://www.nealelab.is/). Additionally, all GWAS data (dataset ID, psoriasis, finn-b-L12_PSORI_VULG; ESAM, prot-a-988; IL-16, ebi-a-GCST004430; AMI, ukb-a-533) in this research are also publicly available at the MRC IEU repository (https://gwas.mrcieu.ac.uk). It is important to note that the datasets used in this study originate from different studies with varying populations, study designs, and conditions. All GWAS datasets are based on European populations, which helps to reduce potential confounding due to population stratification. To minimize the impact of heterogeneity across studies, we employed several strategies: we selected robust genetic instruments, conducted sensitivity analyses (including MR-Egger and weighted median methods) to detect and address pleiotropy and other biases, and used large-scale, well-documented public datasets to ensure consistency and transparency in our analyses. The GWAS datasets included in this study are based on European populations. The current study did not require ethical approval because it was a secondary analysis of public data.

### 2.2. Study design

**Figure 1.**
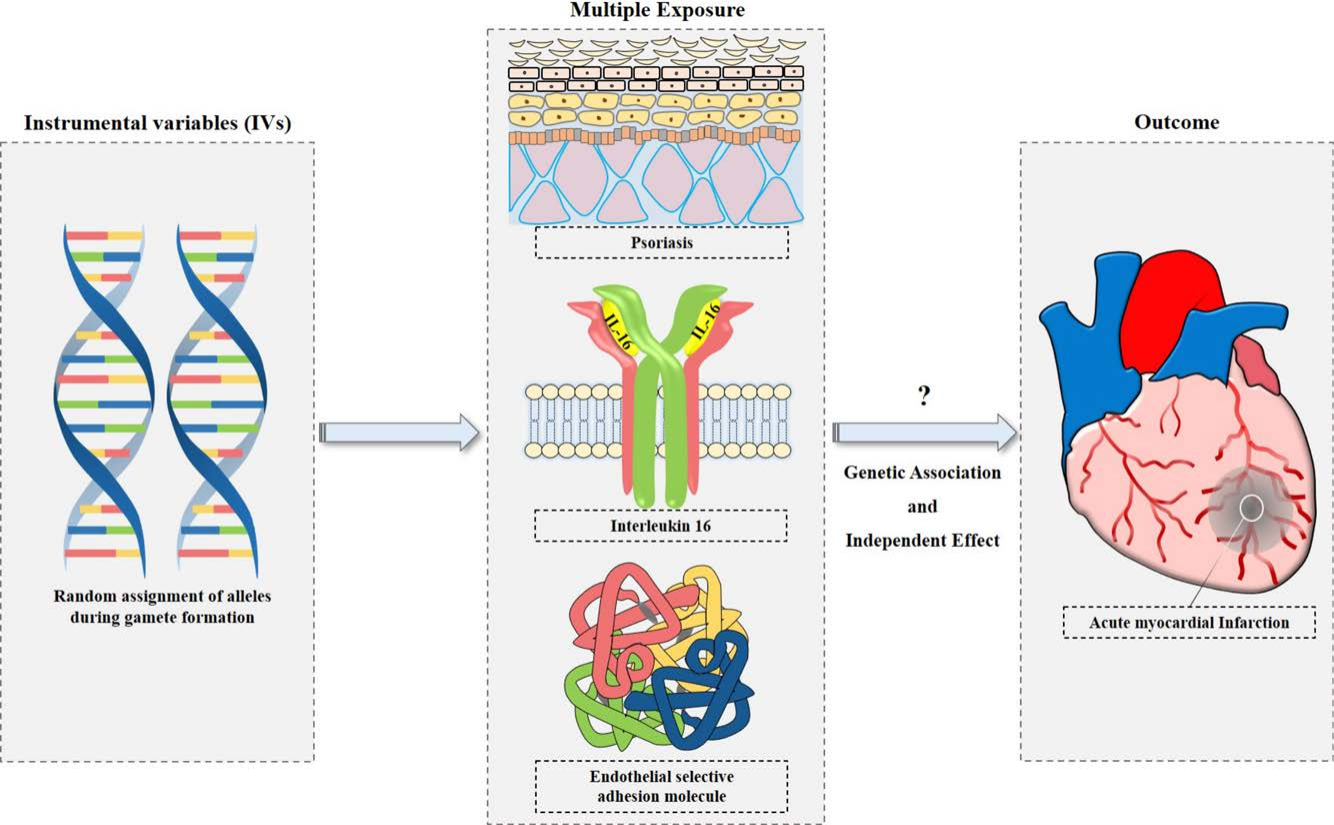
Study hypothesis and flow chart. In the step 1, the 2-sample MR analysis was used to explore the causal effect between psoriasis, ESAM as well as IL-16 and the AMI risk, respectively. In the step 2, the multivariable MR mediation analysis was performed to further assess the degree of ESAM and IL-16 factors mediating the effects of psoriasis on the risk of AMI. AMI = acute myocardial infarction, ESAM = endothelial selective adhesion molecule, IL-16 = interleukin-16, MR = Mendelian randomization.

First, 2-sample MR analysis,^[22]^ a commonly used method, was used to evaluate the causal relationship between psoriasis, ESAM, and IL-16 and the risk of AMI. Single nucleotide polymorphisms (SNPs) were selected as instrumental variables when they simultaneously met the following assumptions: instrumental variables must be strongly correlated with exposures including psoriasis, ESAM, and IL-16; instrumental variables must be independent of several confounding factors; instrumental variables must be only correlated with the risk of AMI via exposures (psoriasis, ESAM, and IL-16). Second, as an extension of univariable MR, the multivariable MR can estimate the independent causal effects of various risk factors jointly on AMI risk by including several exposures such as psoriasis and/or ESAM and/or IL-16 within the same model, and further assess the degree of ESAM and/or IL-16 factors mediating the effects of psoriasis on the AMI risk, respectively. The study hypothesis and flow chart are shown in Figure 1.

### 2.3. Selection of instrumental variables

Selected instrumental variables that are significantly associated with exposures including psoriasis, ESAM, and IL-16 were extracted from the corresponding GWAS using a genome-wide significance threshold of *P *< 5 × 10^−8^. They were considered independent of each other when they met a pairwise linkage disequilibrium *r*^2^ < 0.001, and located kb = 10,000 apart from each other. To avoid horizontal pleiotropy, PhenoScanner^[23]^ was used to further identify and eliminate the SNPs that might be related to confounding factors that can affect AMI. The *F*-statistic was calculated to validate the strength of each SNP based on the *R*^2^ (i.e., the proportion of phenotypic variance explained by each SNP). Then, MR mediation analyses were used to evaluate the degree of ESAM and/or IL-16 factors mediating the effects of genetically-predicted psoriasis on the AMI risk. In multivariable MR analysis, several SNPs that were significantly (*P *< 5 × 10^−8^) associated with psoriasis and/or ESAM and/or IL-16 were extracted from relevant GWAS dataset, and then integrating with existing exposure instrumental variables. After excluding those duplicate SNPs, we obtained the effects and corresponding standard errors for each SNP from the exposures and outcomes. Both the weighted linear regression based inverse variance weighting and MR-Egger approaches were applied to infer causal effects in multivariable MR analysis. All instrumental variables were extracted from GWAS using the TwoSampleMR package in R.^[24]^

### 2.4. Two-sample Mendelian randomization analysis

In our preliminary analysis, the causal effects of psoriasis, ESAM, and IL-16 on the risk of AMI were assessed using the random-effects inverse variance weighting method. The genetic correlation estimates for AMI risk were based on the logistic regression coefficient (i.e., log odds ratio [OR]) of each SNP. The OR estimates provided by MR were obtained by exponentiating the resulting MR estimates. Then, various methods, including MR-Egger, penalized weighted median, simple mode, maximum likelihood, weighted mode, and weighted median, were used to calculate follow-up sensitivity.^[25]^ Compared to the inverse variance weighting, these methods are more robust to individual genetics with strong outlier causal estimates and would produce consistent estimates of causal effects when the effective instrumental variables exceed 50%.^[24]^ Furthermore, the leave-one-out sensitivity analysis was used to assess whether the correlations were significantly affected by a single specific SNP. The Cochran Q test was used to test the heterogeneity between genetic variants. The MR-Egger intercept test was performed to evaluate the horizontal pleiotropy. These analyses were performed using the “TwoSampleMR” R package.^[24]^

### 2.5. Multivariable Mendelian randomization analysis

The multivariable MR mediation analysis was performed to further assess the degree of ESAM and IL-16 factors mediating the effects of psoriasis on the risk of AMI. The total effect of each exposure of interest was divided into direct and indirect effects. Thus, it was possible to assess the potential mediating effects and the proportion of the effect of the primary exposure of interest on the outcome that acts through other considered mediators.^[26,27]^ Specifically, variant-AMI genetic correlation estimates (on the log OR scale) were regressed on variant-psoriasis, variant-ESAM, and variant-IL-16 genetic correlation estimates, and weighted for the precision (i.e., the inverse of their variance) of the variant-AMI genetic correlation estimates and with the intercept fixed at zero.^[28]^ Next, ESAM and IL-16 were incorporated into the model alone or together. The final OR calculation of the effect of psoriasis on AMI risk from the multivariable MR analysis was obtained by exponentiating the corresponding effect estimate. To evaluate the proportion of the effect of genetically-predicted psoriasis on the risk of AMI that was mediated through 2 considered exposures including ESAM and/or IL-16. After adjusting the genetic prediction levels of the ESAM and/or IL-16, the MR estimate for the effect of genetic prediction of psoriasis on AMI risk was divided by the total effect of psoriasis on AMI risk estimated in the inverse variance weighting univariable MR and subtracted from 1. The standard errors were estimated by the propagation of the error method.^[18,29]^

## 3. Results

### 3.1. Selection and validation of SNPs

A total of 8 SNPs were correlated with psoriasis, 3 SNPs were correlated with ESAM, and 3 SNPs were correlated with IL-16, as identified at a genome-wide significance level of *P *< 5 × 10^−8^. The characteristics of these SNPs and their associations with exposures including psoriasis, ESAM, and IL-16, as well as AMI outcome are shown in detail in Table S1, Supplemental Digital Content, https://links.lww.com/MD/P45. All SNPs identified were valid (*F *> 10).

### 3.2. Two-sample Mendelian randomization and multivariable Mendelian randomization analyses

**Figure 2.**
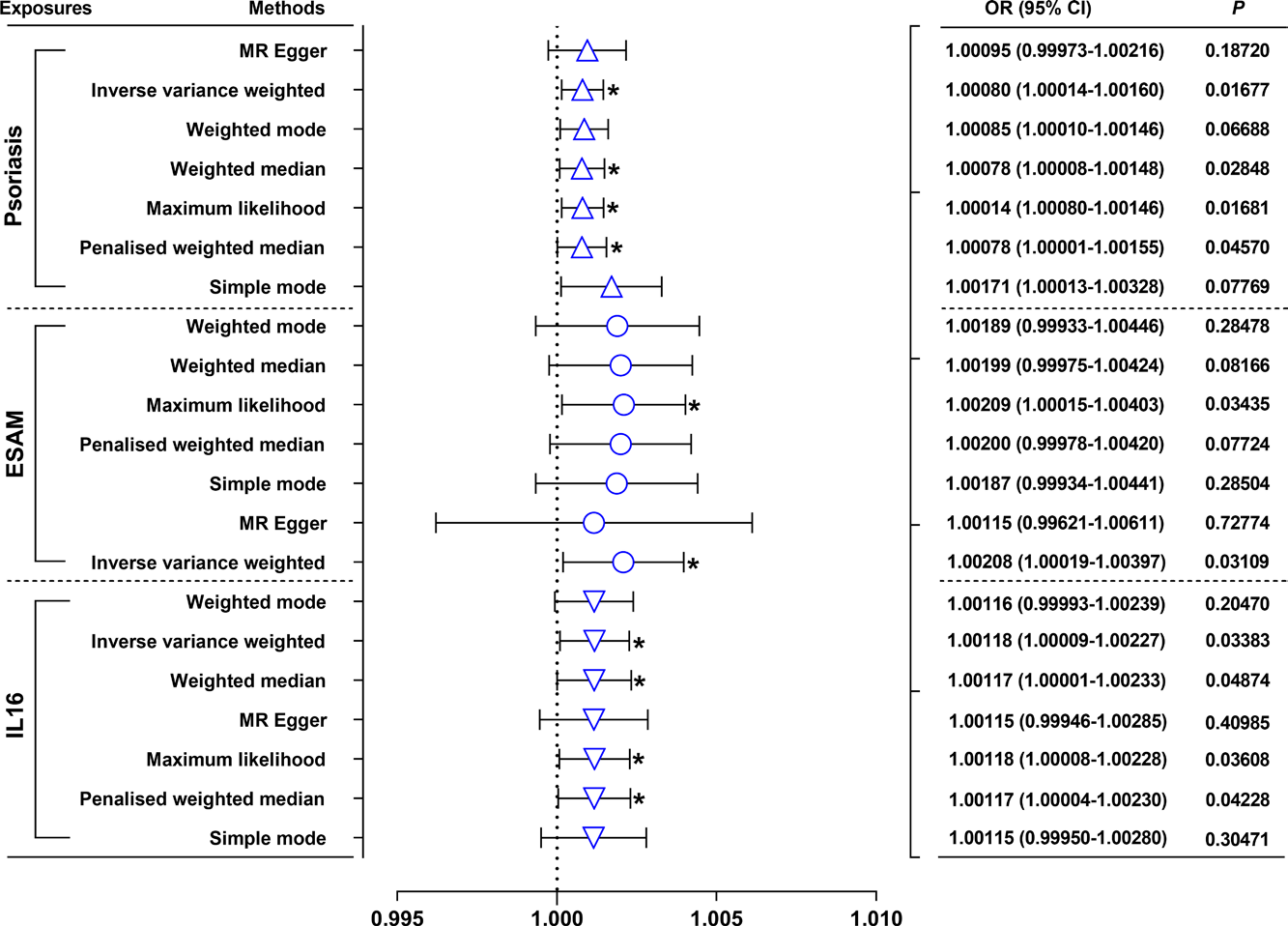
Effects of genetically-predicted psoriasis, ESAM and IL-16 respectively on AMI risk. AMI = acute myocardial infarction, CI = confidence interval, ESAM = endothelial selective adhesion molecule, IL-16 = interleukin-16, MR = Mendelian randomization, OR = odds ratio.

The random-effects inverse variance weighting analysis showed that psoriasis (OR = 1.00078; 95% confidence interval [CI]: 1.00008–1.00148; *P *= .028479), ESAM (OR = 1.00208; 95% CI: 1.00019–1.00397; *P *= .031089), and IL-16 (OR = 1.00118, 95% CI: 1.00009–1.00227; *P *= .033826) were risk factors for AMI (Fig. 2 and Table S2, Supplemental Digital Content, https://links.lww.com/MD/P45). Scatter plots showed that psoriasis, ESAM, and IL-16 were positively associated with AMI incidence (Fig. S1, Supplemental Digital Content, https://links.lww.com/MD/P46). The sensitivity analysis using the leave-one-out method also confirmed the causal effect between psoriasis, ESAM, as well as IL-16 and AMI (Fig. S2, Supplemental Digital Content, https://links.lww.com/MD/P46). No heterogeneity (heterogeneity test, Table S3, Supplemental Digital Content, https://links.lww.com/MD/P45) or horizontal pleiotropy (MR-Egger intercept test, Table S4, Supplemental Digital Content, https://links.lww.com/MD/P45) were detected in the 2-sample MR analysis. The overall estimates, as calculated by MR-Egger or inverse variance weighting tests, also revealed the causal effect between psoriasis, ESAM, as well as IL-16 and AMI (Fig. S3, Supplemental Digital Content, https://links.lww.com/MD/P46).

**Figure 3.**
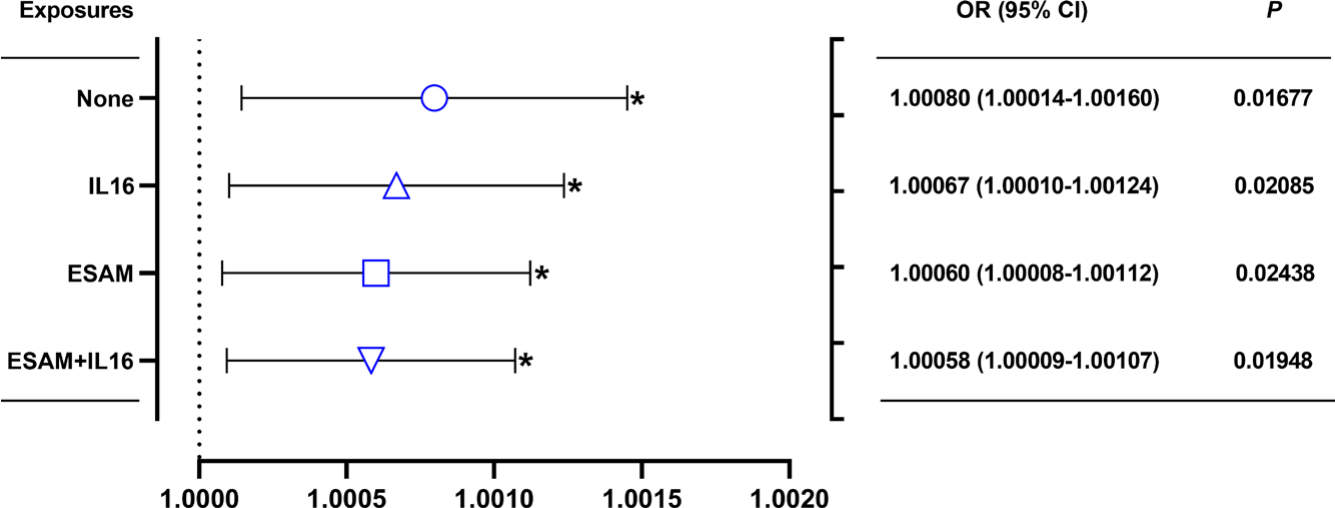
The effect of genetically-predicted psoriasis on AMI risk after adjusting for genetically-predicted ESAM and IL-16, either separately or in the same model. The y-axis details the adjustment made. AMI = acute myocardial infarction, CI = confidence interval, ESAM = endothelial selective adhesion molecule, IL-16 = interleukin-16, OR = odds ratio.

**Figure 4.**
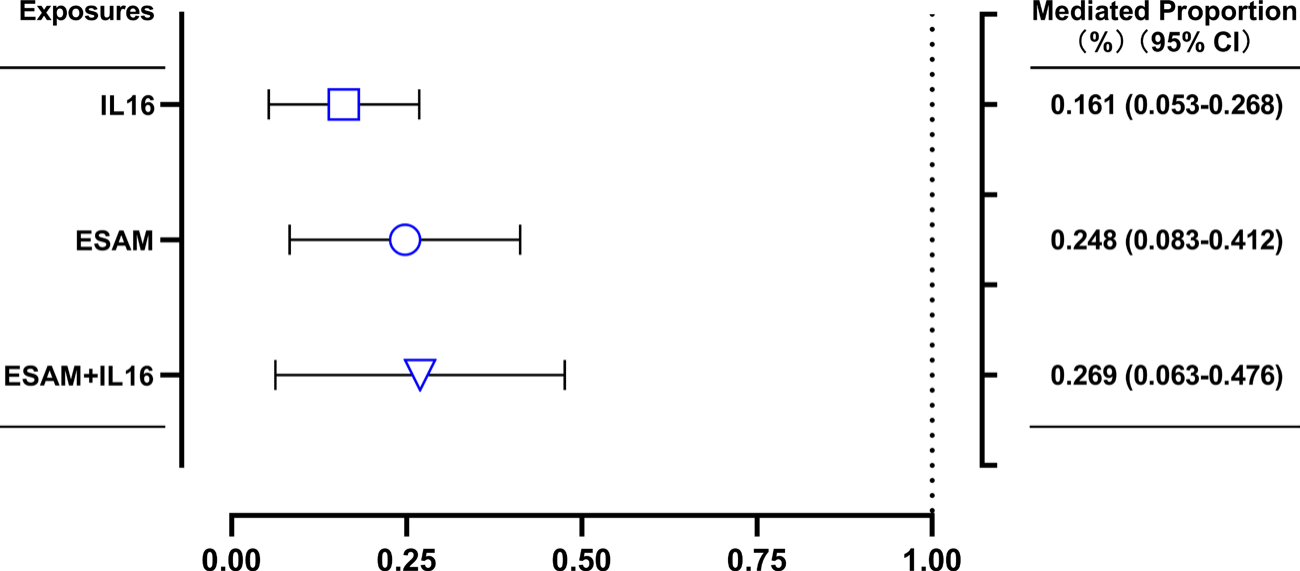
The percentage of the effect of genetically-predicted psoriasis on AMI risk that is mediated through genetically-predicted ESAM and IL-16, separately and when considered together in the same model. The y-axis details the mediating pathway considered AMI = acute myocardial infarction, ESAM = endothelial selective adhesion molecule, IL-16 = interleukin-16.

The multivariable MR mediation analysis was used to evaluate the degree to which these 2 traits (ESAM and IL-16) mediated the effects of genetically-predicted psoriasis on AMI risk. In the multivariable MR analysis, the pathogenic effects of genetically-predicted psoriasis on AMI risk attenuated the OR (OR = 1.00080; 95% CI: 1.00014–1.00145; *P *= .016773) in inverse variance weighting univariable analysis to the OR of 1.00060 (95% CI: 1.00008–1.00112; *P* = .024380) after adjusting for genetically-predicted ESAM; to the OR of 1.0067 (95% CI: 1.00010–1.00124; *P* = .020854) after adjusting for genetically-predicted IL-16; and to the OR of 1.00058 (95% CI: 1.00009–1.00107; *P* = .019478) after adjusting for both genetically-predicted ESAM and IL-16 (Fig. 3). These results suggested that ESAM and IL-16 play a mediating role in the effect of psoriasis on the AMI risk. The proportion of the effects of genetically-predicted psoriasis mediated by genetically-predicted ESAM, IL-16, and both ESAM and IL-16 was estimated as 24.8% (95% CI: 8.3%–41.2%), 16.1% (95% CI: 5.3%–26.8%), and 26.9% (95% CI: 6.3%–47.6%), respectively (Fig. 4).

## 4. Discussion

Psoriasis is an inflammatory skin disease that can cause a chronic systemic inflammatory response. Chronic systemic inflammatory response combined with the increased prevalence of traditional cardiovascular risk factors will lead to endothelial dysfunction,^[30]^ comprehending the initial step in the occurrence and development of atherosclerosis and its cardiovascular complications.^[31]^ In 1973, it was first reported that psoriasis patients have twice the risk of major adverse cardiovascular events compared to patients suffering from other skin diseases.^[32]^ A meta-analysis involving 14 cohort studies further indicated that severe psoriasis patients have a significantly increased risk of cardiovascular diseases.^[33]^ Increasing evidence has supported that several inflammatory biomarkers, such as chemokines, cell adhesion molecules, C-reactive protein, and cytokines (i.e. Tumour necrosis factor-α and IL-17) are associated with an increased risk of atherosclerosis and cardiovascular diseases in psoriasis patients.^[34,35]^ Psoriasis has been considered as an independent risk factor for coronary heart disease^[36]^ and AMI.^[37,38]^ Our current findings are consistent with these studies. To the best of our knowledge, this is the first study to report the causal correlation between genetically-predicted psoriasis and the risk of AMI using MR analysis. Further, we quantified the mediating effects of genetically-predicted ESAM and Il-16 among the effects of psoriasis on AMI risk. These findings might help understand the underlying inflammation-related molecular mechanisms of psoriasis prone to AMI.

As a cell adhesion molecule, ESAM is a member of the newly discovered immunoglobulin superfamily, which is highly expressed in vascular endothelial cells.^[39]^ Wegmann et al have shown that ESAM can effectively enhance neutrophil extravasation, Rho activation, and vascular permeability induced by VEGF.^[5]^ Inoue et al have found that ESAM can affect vascular endothelial function by mediating endothelial permeability and leukocyte migration. The inactivation of ESAM can also effectively reduce the susceptibility to atherosclerosis by inhibiting plaque neovascularization and macrophage infiltration into the atheroma.^[6]^ Park et al have shown that sESAM can be used as a biomarker of endothelial dysfunction and a risk factor for renal dysfunction and albuminuria in coronary artery disease patients.^[40]^ Additionally, Rohatgi et al have suggested that sESAM is independently associated with abdominal aortic wall thickness, coronary artery calcification, and vascular stiffness.^[7]^ Higher levels of sESAM in patients suffering from stable ischemic heart disease were correlated with an increased incidence of composite primary endpoints, including heart failure, myocardial infarction, and death. However, this association was attenuated after adjusting for eGFR.^[41]^ Nevertheless, there is still a lack of direct evidence showing the causal relationship between ESAM and AMI. Herein, we indicated the causal association between genetically-predicted ESAM and the AMI risk. The proportion of the effecs of genetically-predicted psoriasis mediated by genetically-predicted ESAM was 24.8% (95% CI 8.3–41.2%). These results suggested that ESAM plays a partial mediating role during psoriasis complicated with AMI.

Previous studies have shown that the proinflammatory cytokine IL-16 can effectively amplify the inflammatory process and has an immunomodulatory function.^[42]^ The activation of IL-16 can also aggravate infectious, immune-mediated, and autoimmune inflammatory diseases, including viral infections, systemic lupus erythematosus, irritable bowel syndrome, atopic dermatitis, and neurodegenerative diseases.^[43]^ Kawabata et al have shown that IL-16 can be used as a biomarker to evaluate the severity of skin sclerosis. They also showed that inhibiting the activation of IL-16 might be an effective treatment for systemic sclerosis.^[44]^ Purzycka-Bohdan et al have found that the serum levels of IL-16 in psoriasis patients are significantly higher than those in healthy controls. The serum levels of IL-16 are also positively correlated with the psoriatic area and severity index score, indicating that IL-16 is a potential marker for evaluating the activity of psoriasis.^[45]^ Moreover, Park et al have suggested that IL-16 can induce the migration and invasion of vascular smooth muscle cells through the p38MAPK/Sp-1/MMP-9 pathway. Thus, IL-16 might become a new target for the treatment of vascular diseases, including atherosclerosis and restenosis.^[46]^ However, Grönberg et al have found that elevated circulating levels of IL-16 contribute to the stability of carotid plaque and are associated with a reduced risk of new cardiovascular events within 2 years after endarterectomy. These findings suggested that IL-16 might also play a protective role in human atherosclerotic diseases.^[47]^ Overall, the above findings indicated that whether IL-16 can be a promoter or suppressor in atherosclerosis and its related diseases remain controversial. First, observational studies are not enough to detect the true correlation due to the limitation of sample size. Moreover, in observational studies, the regression approach might not provide unbiased estimates of true associations when confounding factors cannot be distinguished, since they are usually unknown or unmeasurable, or when the number of confounding factors is too large. Since genetic variations are randomly assigned at conception, their correlations with outcomes are rarely affected by environmental confounding factors. Thus, the MR approach based on genetic variations as instrumental variables is often used to explore causality.^[48,49]^ Here, we found a causal correlation between genetically-predicted IL-16 and AMI risk. Additionally, the proportion of the effects of genetically-predicted psoriasis mediated by genetically-predicted IL-16 was 16.1% (95% CI 5.3%–26.8%). These results indicate d that IL-16 also plays a partial mediating role during psoriasis complicated with AMI.

Our current study also has some limitations. First, the GWAS datasets of psoriasis, AMI, and ESAM contain male and female samples, while the GWAS dataset of IL-16 lacks gender information. Hence, there might be gender differences among research participants that were not evaluated. Second, the participants in this study are of European descent, which might also limit the expansion of our findings to other ethnicities. Third, the key to mediation analysis depends on the correct formulation of the causal exposure correlations, because mediation and confusion are statistically indistinguishable.^[50]^ Fourth, interpreting mediation analysis results for a binary outcome is not straightforward due to the non-collapsibility of the OR, as the estimate of the mediation proportion might be biased.^[22]^ Fifth, we only found the mediating role of ESAM and IL-16 in psoriasis complicated with AMI from the perspective of genetics. However, the role of other inflammatory factors in this process remains unclear and should be explored in the future.

## 5. Conclusions

The MR analysis supported that psoriasis, ESAM, and IL-16 are risk factors for AMI. Additionally, ESAM and IL-16 play an intermediary role in psoriasis complicated with AMI. The targeted intervention of ESAM and IL-16 might help decrease the risk of AMI in psoriasis patients.

## Acknowledgments

The authors thank the studies reported by Sun et al and Ahola-Olli et al or consortiums including the FinnGen study and the Neale Lab Consortium that mentioned and included in the present analysis for providing public datasets.

## Author contributions

**Conceptualization:** Hai-Jun Zhang.

**Data curation:** Chao Wang.

**Funding acquisition:** Xuan Kang, Hai-Jun Zhang.

**Methodology:** Xuan Kang, Hai-Jun Zhang.

**Visualization:** Chao Wang.

**Writing – original draft:** Xuan Kang.

**Writing – review & editing:** Guo-Qiang Zhong, Hai-Jun Zhang.

## Supplementary Material



## References

[1] Wolf D Ley K. Immunity and inflammation in atherosclerosis. Circ Res. 2019;124:315–27. 30653442 10.1161/CIRCRESAHA.118.313591 PMC6342482

[2] Lockshin B Balagula Y Merola JF. Interleukin 17, inflammation, and cardiovascular risk in patients with psoriasis. J Am Acad Dermatol. 2018;79:345–52. 29477740 10.1016/j.jaad.2018.02.040

[3] Armstrong AW Harskamp CT Ledo L Rogers JH Armstrong EJ. Coronary artery disease in patients with psoriasis referred for coronary angiography. Am J Cardiol. 2012;109:976–80. 22221950 10.1016/j.amjcard.2011.11.025

[4] Golden JB McCormick TS Ward NL. IL-17 in psoriasis: implications for therapy and cardiovascular co-morbidities. Cytokine. 2013;62:195–201. 23562549 10.1016/j.cyto.2013.03.013 PMC3640599

[5] Wegmann F Petri B Khandoga AG. ESAM supports neutrophil extravasation, activation of Rho, and VEGF-induced vascular permeability. J Exp Med. 2006;203:1671–7. 16818677 10.1084/jem.20060565 PMC2118342

[6] Inoue M Ishida T Yasuda T. Endothelial cell-selective adhesion molecule modulates atherosclerosis through plaque angiogenesis and monocyte-endothelial interaction. Microvasc Res. 2010;80:179–87. 20406651 10.1016/j.mvr.2010.04.005

[7] Rohatgi A Owens AW Khera A. Differential associations between soluble cellular adhesion molecules and atherosclerosis in the Dallas Heart Study: a distinct role for soluble endothelial cell-selective adhesion molecule. Arterioscler Thromb Vasc Biol. 2009;29:1684–90. 19759376 10.1161/ATVBAHA.109.190553 PMC2771407

[8] Brunner PM Suárez-Fariñas M He H. The atopic dermatitis blood signature is characterized by increases in inflammatory and cardiovascular risk proteins. Sci Rep. 2017;7:8707. 28821884 10.1038/s41598-017-09207-z PMC5562859

[9] Schernthaner C Paar V Wernly B. Elevated plasma levels of interleukin-16 in patients with acute myocardial infarction. Medicine (Baltimore). 2017;96:e8396. 29095267 10.1097/MD.0000000000008396 PMC5682786

[10] Roudbar MA Mohammadabadi MR Mehrgardi AA. Integration of single nucleotide variants and whole-genome DNA methylation profiles for classification of rheumatoid arthritis cases from controls. Heredity (Edinb). 2020;124:658–74. 32127659 10.1038/s41437-020-0301-4 PMC7171157

[11] Barazandeh A Mohammadabadi M Ghaderi-Zefrehei M Nezamabadi-Pour H. Genome-wide analysis of CpG islands in some livestock genomes and their relationship with genomic features original paper. Czech J Animal Sci. 2016;61:487–95.

[12] Alavi M Mozafari MR Ghaemi S Ashengroph M Hasanzadeh Davarani F Mohammadabadi M. Interaction of epigallocatechin gallate and quercetin with Spike Glycoprotein (S-Glycoprotein) of SARS-CoV-2: In Silico Study. Biomedicines. 2022;10:3074. 36551830 10.3390/biomedicines10123074 PMC9775955

[13] Heidarpour F Mohammadabadi MR Zaidul IS. Use of prebiotics in oral delivery of bioactive compounds: a nanotechnology perspective. Pharmazie. 2011;66:319–24. 21699064

[14] Khabiri A Toroghi R Mohammadabadi M Tabatabaeizadeh SE. Introduction of a Newcastle disease virus challenge strain (sub-genotype VII.1.1) isolated in Iran. Vet Res Forum. 2023;14:221–8. 37181855 10.30466/vrf.2022.548152.3373 PMC10170471

[15] Safaei SMH Dadpasand M Mohammadabadi M. An origanum majorana leaf diet influences myogenin gene expression, performance, and carcass characteristics in lambs. Animals (Basel). 2022;13:14. 36611623 10.3390/ani13010014 PMC9817680

[16] Bordbar F Mohammadabadi M Jensen J Xu L Li J Zhang L. Identification of candidate genes regulating carcass depth and hind leg circumference in simmental beef cattle using illumina bovine beadchip and next-generation sequencing analyses. Animals (Basel). 2022;12:1103. 35565529 10.3390/ani12091103 PMC9102740

[17] Lawlor DA Harbord RM Sterne JA Timpson N Davey Smith G. Mendelian randomization: using genes as instruments for making causal inferences in epidemiology. Stat Med. 2008;27:1133–63. 17886233 10.1002/sim.3034

[18] Burgess S Thompson DJ Rees JMB Day FR Perry JR Ong KK. Dissecting causal pathways using mendelian randomization with summarized genetic data: application to age at menarche and risk of breast cancer. Genetics. 2017;207:481–7. 28835472 10.1534/genetics.117.300191 PMC5629317

[19] Holmes MV Ala-Korpela M Smith GD. Mendelian randomization in cardiometabolic disease: challenges in evaluating causality. Nat Rev Cardiol. 2017;14:577–90. 28569269 10.1038/nrcardio.2017.78 PMC5600813

[20] Sun BB Maranville JC Peters JE. Genomic atlas of the human plasma proteome. Nature. 2018;558:73–9. 29875488 10.1038/s41586-018-0175-2 PMC6697541

[21] Ahola-Olli AV Würtz P Havulinna AS. Genome-wide association study identifies 27 loci influencing concentrations of circulating cytokines and growth factors. Am J Hum Genet. 2017;100:40–50. 27989323 10.1016/j.ajhg.2016.11.007 PMC5223028

[22] Hartwig FP Davies NM Hemani G Davey Smith G. Two-sample Mendelian randomization: avoiding the downsides of a powerful, widely applicable but potentially fallible technique. Int J Epidemiol. 2016;45:1717–26. 28338968 10.1093/ije/dyx028 PMC5722032

[23] Staley JR Blackshaw J Kamat MA. PhenoScanner: a database of human genotype-phenotype associations. Bioinformatics (Oxford, England). 2016;32:3207–9. 27318201 10.1093/bioinformatics/btw373 PMC5048068

[24] Hemani G Zheng J Elsworth B. The MR-Base platform supports systematic causal inference across the human phenome. eLife. 2018;7:e34408. 29846171 10.7554/eLife.34408 PMC5976434

[25] Benn M Nordestgaard BG. From genome-wide association studies to Mendelian randomization: novel opportunities for understanding cardiovascular disease causality, pathogenesis, prevention, and treatment. Cardiovasc Res. 2018;114:1192–208. 29471399 10.1093/cvr/cvy045

[26] Burgess S Thompson SG. Multivariable Mendelian randomization: the use of pleiotropic genetic variants to estimate causal effects. Am J Epidemiol. 2015;181:251–60. 25632051 10.1093/aje/kwu283 PMC4325677

[27] Sanderson E. Multivariable mendelian randomization and mediation. Cold Spring Harbor Perspectives Med. 2021;11:a038984. 10.1101/cshperspect.a038984 PMC7849347 32341063

[28] Burgess S Dudbridge F Thompson SG. Re: “Multivariable Mendelian randomization: the use of pleiotropic genetic variants to estimate causal effects.”. Am J Epidemiol. 2015;181:290–1. 25660081 10.1093/aje/kwv017

[29] Carter AR Sanderson E Hammerton G. Mendelian randomisation for mediation analysis: current methods and challenges for implementation. Eur J Epidemiol. 2021;36:465–78. 33961203 10.1007/s10654-021-00757-1 PMC8159796

[30] Puig L. Cardiometabolic comorbidities in psoriasis and psoriatic arthritis. Int J Mol Sci . 2017;19:58. 29295598 10.3390/ijms19010058 PMC5796008

[31] Wierzbowska-Drabik K Lesiak A Skibińska M Niedźwiedź M Kasprzak JD Narbutt J. Psoriasis and atherosclerosis-skin, joints, and cardiovascular story of two plaques in relation to the treatment with biologics. Int J Mol Sci . 2021;22:10402. 34638740 10.3390/ijms221910402 PMC8508744

[32] McDonald CJ Calabresi P. Occlusive vascular disease in psoriatic patients. New England J Med. 1973;288:912. 4692910

[33] Samarasekera EJ Neilson JM Warren RB Parnham J Smith CH. Incidence of cardiovascular disease in individuals with psoriasis: a systematic review and meta-analysis. J Invest Dermatol. 2013;133:2340–6. 23528816 10.1038/jid.2013.149

[34] Siegel D Devaraj S Mitra A Raychaudhuri SP Raychaudhuri SK Jialal I. Inflammation, atherosclerosis, and psoriasis. Clin Rev Allergy Immunol. 2013;44:194–204. 22359071 10.1007/s12016-012-8308-0

[35] Boehncke WH. Systemic inflammation and cardiovascular comorbidity in psoriasis patients: causes and consequences. Front Immunol. 2018;9:579. 29675020 10.3389/fimmu.2018.00579 PMC5895645

[36] Kim J Tomalin L Lee J. Reduction of inflammatory and cardiovascular proteins in the blood of patients with psoriasis: differential responses between tofacitinib and etanercept after 4 weeks of treatment. J Invest Dermatol. 2018;138:273–81. 28927890 10.1016/j.jid.2017.08.040

[37] Lin HW Wang KH Lin HC Lin HC. Increased risk of acute myocardial infarction in patients with psoriasis: a 5-year population-based study in Taiwan. J Am Acad Dermatol. 2011;64:495–501. 21216492 10.1016/j.jaad.2010.01.050

[38] Shiba M Kato T Izumi T. Risk of myocardial infarction in patients with psoriasis: a cross-sectional patient-population study in a Japanese hospital. J Cardiol. 2019;73:276–9. 30583988 10.1016/j.jjcc.2018.10.008

[39] Hirata K Ishida T Penta K. Cloning of an immunoglobulin family adhesion molecule selectively expressed by endothelial cells. J Biol Chem. 2001;276:16223–31. 11279107 10.1074/jbc.M100630200

[40] Park M Vittinghoff E Ganz P Peralta CA Whooley M Shlipak MG. Role of soluble endothelial cell-selective adhesion molecule biomarker in albuminuria and kidney function changes in patients with coronary artery disease: the Heart and Soul Study. Arterioscler Thromb Vasc Biol. 2014;34:231–6. 24177327 10.1161/ATVBAHA.113.301806 PMC4059045

[41] Park M Kulkarni A Beatty A. Soluble endothelial cell selective adhesion molecule and cardiovascular outcomes in patients with stable coronary disease: a report from the Heart and Soul Study. Atherosclerosis. 2015;243:546–52. 26523992 10.1016/j.atherosclerosis.2015.10.092 PMC4663109

[42] Nagy G Gáspár K Irinyi B. Association between serum IL-16 levels and the degree of sensitization in patients with atopic dermatitis. Int Arch Allergy Immunol. 2011;156:69–74. 21447961 10.1159/000321959

[43] Glass WG Sarisky RT Vecchio AM. Not-so-sweet sixteen: the role of IL-16 in infectious and immune-mediated inflammatory diseases. J Interferon Cytokine Res. 2006;26:511–20. 16881862 10.1089/jir.2006.26.511

[44] Kawabata K Makino T Makino K Kajihara I Fukushima S Ihn H. IL-16 expression is increased in the skin and sera of patients with systemic sclerosis. Rheumatology (Oxford). 2020;59:519–23. 31377804 10.1093/rheumatology/kez318

[45] Purzycka-Bohdan D Szczerkowska-Dobosz A Zablotna M. Assessment of interleukin 16 serum levels and skin expression in psoriasis patients in correlation with clinical severity of the disease. PLoS One. 2016;11:e0165577. 27788245 10.1371/journal.pone.0165577 PMC5082815

[46] Park SL Hwang B Lee SY. p21WAF1 is required for interleukin-16-induced migration and invasion of vascular smooth muscle cells via the p38MAPK/Sp-1/MMP-9 pathway. PLoS One. 2015;10:e0142153. 26544695 10.1371/journal.pone.0142153 PMC4636239

[47] Grönberg C Bengtsson E Fredrikson GN. Human carotid plaques with high levels of interleukin-16 are associated with reduced risk for cardiovascular events. Stroke. 2015;46:2748–54. 26330445 10.1161/STROKEAHA.115.009910

[48] Davey Smith G Hemani G. Mendelian randomization: genetic anchors for causal inference in epidemiological studies. Hum Mol Genet. 2014;23:R89–98. 25064373 10.1093/hmg/ddu328 PMC4170722

[49] Ference BA Holmes MV Smith GD. Using mendelian randomization to improve the design of randomized trials. Cold Spring Harbor Perspectives Med. 2021;11:a040980. 10.1101/cshperspect.a040980 PMC8247560 33431510

[50] MacKinnon DP Krull JL Lockwood CM. Equivalence of the mediation, confounding and suppression effect. Prevention Sci. 2000;1:173–81. 10.1023/a:1026595011371 PMC2819361 11523746

