# Supplementary material for: Endothelial selective adhesion molecule and interleukin-16 play an intermediary role in psoriasis complicated with acute myocardial infarction: A Mendelian randomization study
Source: Medicine (Baltimore). 2025 May 23;104(21):e42538. doi: 10.1097/MD.0000000000042538 (PMC12113922; doi:10.1097/MD.0000000000042538)

Supplementary Figure 1. Scatter plots of the estimated SNP effects on exposures (x-axis) plotted against the estimated SNPs effects on the AMI (y-axis). A, Psoriasis; B, ESAM; C, IL-16. The slope of the line corresponds to a causal estimate using a different method.

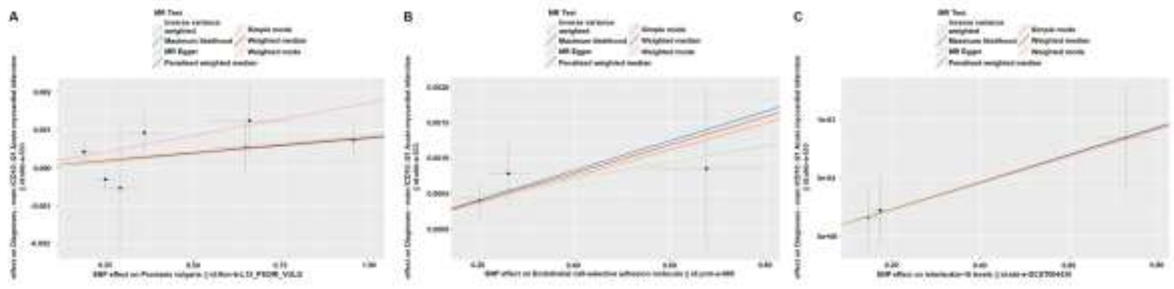

Supplementary Figure 2. Sensitivity analyses using the leave-one-out approach on the association of exposures on AMI. A, Psoriasis; B, ESAM; C, IL-16. Each black dot represents an IVW method for estimating causal the effect of the exposures on the AMI does not exclude a case where a particular SNP caused a significant change in the overall results.

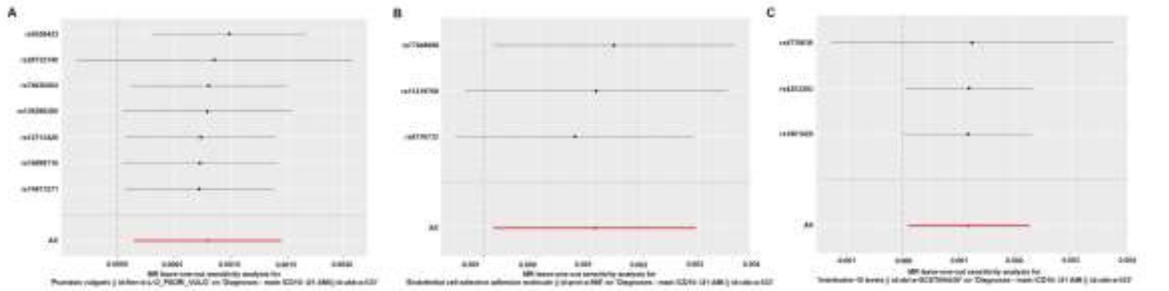

Supplementary Figure 3. Results of the single and multi SNP analyses for the SNP effect of exposures on CKD. A, Psoriasis; B, ESAM; C, IL-16. The forest map, where each black dot represented a single SNP as IV, showed the logarithm of the odds the ratio 95% (OR) confidence per standard deviation under the influence of exposures; the red dot showed the use of IVW results for all SNPs; the horizontal line indicated the 95% confidence interval.

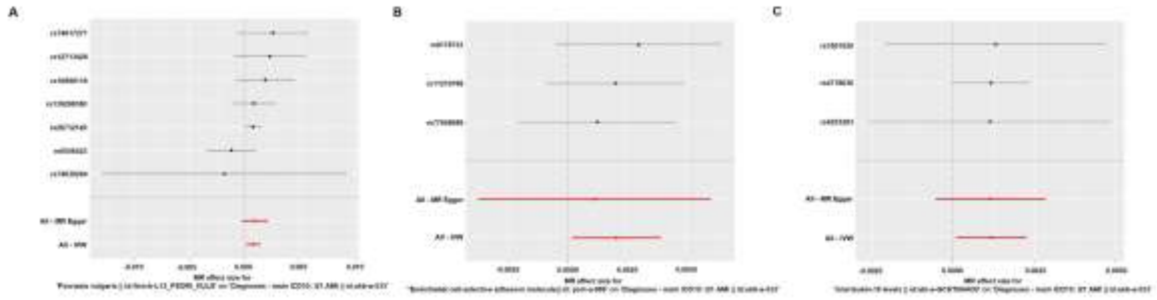

Supplement: Supplementary file 2 [file medi-104-e42538-s002.pdf]
